# Supplementary material for: HLA-G Alleles Impact the Perinatal Father–Child HPV Transmission
Source: Curr Issues Mol Biol. 2023 Jul 12;45(7):5798–810. doi: 10.3390/cimb45070366 (PMC10378566; doi:10.3390/cimb45070366)
Supplement: Supplementary file 1 [file cimb-45-00366-s001.zip › cimb-2472083-supplementary.pdf]

HLA-G alleles impact the perinatal father-child HPV transmission

Nelli T. Suominen, Michel Roger, Marie-Claude Faucher, Kari J. Syrjänen, Seija E. Grénman, Stina M. Syrjänen, Karolina Louvanto

Corresponding author: Karolina Louvanto

Table S1 Association of Human leukocyte antigen G (HLA-G) allele sharing with postpartum Human papillomavirus (HPV) prevalence among 134 father-child pairs of the Finnish Family HPV study. The father-child pairs with both parties being negative for these specific alleles served as a reference in high resolution HLA-allele groups. In low resolution HLA-G allele groups discordant father-child pairs served as a reference.

		HPV: father semen/child oral[b] OR (95 % CI)			HPV: farther urethra/child oral[b] OR (95 % CI)			HPV: father oral/child oral[b] OR (95 % CI)		
HLA-G allele concordance[a]	allele sharing	any	LR	HR	any	LR	HR	any	LR	HR
***01:01:01**	0	1.00	1.00	1.00	1.00	1.00	1.00	1.00	1.00	1.00
	1	NA	NA	NA	NA	NA	NA	NA	NA	NA
	≥2	NA	NA	NA	NA	NA	NA	NA	NA	NA
***01:01:02**	0	1.00	1.00	1.00	1.00	1.00	1.00	1.00	1.00	1.00
discordance	1	1.88 (0.81-4.34)	1.89 (0.62-5.74)	1.22 (0.53-2.81)	1.81 (0.77-4.26)	0.96 (0.31-3.02)	1.55 (0.67-3.58)	2.33 (0.98-5.53)	1.92 (0.60-6.16)	1.64 (0.71-3.80)
concordance	≥2	2.32 (0.94-5.68)	2.39 (0.80-7.08)	1.06 (0.44-2.54)	1.54 (0.64-3.69)	0.68 (0.20-2.28)	1.27 (0.52-3.09)	1.14 (0.48-2.70)	1.23 (0.34-4.47)	0.76 (0.31-1.88)
***01:01:03**	0	1.00	1.00	1.00	1.00	1.00	1.00	1.00	1.00	1.00
discordance	1	0.52 (0.14-1.92)	0.69 (0.40-7.14)	0.70 (0.19-2.62)	1.31 (0.35-4.87)	NA	1.85 (0.50-6.92)	0.40 (0.10-1.62)	NA	0.59 (0.14-2.38)
concordance	≥2	0.15 (0.02-1.36)	0.79 (0.09-7.15)	0.21 (0.02-1.86)	0.87 (0.17-4.50)	0.86 (0.10-7.73)	1.24 (0.24-6.38)	1.87 (0.33-10.59)	1.07 (0.12-9.75)	2.73 (0.48-15.53)
***01:01:14**	0	1.00	1.00	1.00	1.00	1.00	1.00	1.00	1.00	1.00
discordance	1	NA	NA	NA	NA	NA	NA	NA	NA	NA
concordance	≥2	0.43 (0.04-4.92)	NA	0.58 (0.05-6.52)	1.76 (0.16-19.95)	5.05 (0.30-84.13)	0.57 (0.05-6.42)	0.48 (0.04-5.48)	NA	0.66 (0.06-7.51)
***01:03:01**	0	1.00	1.00	1.00	1.00	1.00	1.00	1.00	1.00	1.00
discordance	1	1.32 (0.21-8.17)	NA	1.71 (0.28-10.60)	1.34 (0.22-8.29)	NA	1.80 (0.29-11.16)	1.48 (0.24-9.14)	NA	2.05 (0.33-12.70)
concordance	≥2	0.88 (0.12-6.44)	1.83 (0.16-21.04)	0.38 (0.04-3.76)	2.68 (0.27-26.45)	1.49 (0.15-15.06)	1.20 (0.16-8.80)	0.98 (0.13-7.21)	1.92 (0.19-19.57)	0.46 (0.05-4.50)
***01:04:01**	0	1.00	1.00	1.00	1.00	1.00	1.00	1.00	1.00	1.00
discordance	1	0.40 (0.15-1.08)	0.63 (0.17-2.35)	0.44 (0.16-1.23)	0.43 (0.16-1.18)	0.48 (0.10-2.24)	0.62 (0.23-1.69)	0.65 (0.25-1.73)	0.28 (0.04-2.29)	0.98 (0.37-2.60)
concordance	≥2	2.14 (0.54-8.55)	NA	2.95 (0.74-11.78)	2.14 (0.54-8.55)	0.51 (0.06-4.30)	2.02 (0.55-7.33)	2.61 (0.66-10.42)	0.85 (0.10-7.58)	2.56 (0.70-9.31)
***01:06**	0	1.00	1.00	1.00	1.00	1.00	1.00	1.00	1.00	1.00
discordance	1	0.43 (0.08-2.41)	0.76 (0.08-6.88)	0.22 (0.03-1.94)	0.40 (0.07-2.29)	2.53 (0.43-14.79)	0.22 (0.02-1.91)	0.48 (0.08-2.70)	1.24 (0.14-11.29)	0.26 (0.03-2.25)
concordance	≥2	1.70 (0.15-19.26)	1.91 (0.17-22.03)	2.21 (0.19-24.98)	0.40 (0.04-4.58)	2.53 (0.22-29.29)	0.54 (0.05-6.15)	1.90 (0.17-21.56)	3.09 (0.26-36.13)	0.64 (0.06-7.23)
Low resolution										
***01:01+**	1	1.00	1.00	1.00	1.00	1.00	1.00	1.00	1.00	1.00
	2	8.25 (0.65-104.19)	NA	6.00 (0.49-73.45)	2.00 (0.21-18.69)	NA	1.14 (0.13-10.39)	8.25 (0.65-104.19)	NA	3.43 (0.29-40.95)
	3	2.63 (0.24-28.20)	NA	1.50 (0.14-16.32)	0.71 (0.09-5.73)	NA	0.56 (0.07-4.57)	2.80 (0.26-30.18)	NA	2.12 (0.20-22.90)
	4	3.46 (0.35-34.64)	NA	2.86 (0.29-28.62)	1.27 (0.17-9.45)	NA	0.93 (0.13-6.92)	2.86 (0.29-28.62)	NA	2.14 (0.21-21.47)

[a]Only those alleles that were ≥3 % prevalent were included in the analyses.

[b]Father's semen, urethral or oral HPV prevalence at baseline and/or child's oral postpartum HPV prevalence.

0 = no shared alleles; 1 = discordant for the allele; ≥2 = at least 2 shared alleles i.e. both heterozygous or homozygous for the allele; 2 = 2 shared alleles, 3 = 3 shared alleles, 4 = 4 shared alleles

NA=not applicable.
